# Development and Psychometric Properties of the Test of Passive Aggression

**DOI:** 10.3389/fpsyg.2021.579183

**Published:** 2021-04-26

**Authors:** Christian G. Schanz, Monika Equit, Sarah K. Schäfer, Michael Käfer, Hannah K. Mattheus, Tanja Michael

**Affiliations:** ^1^Clinical Psychology and Psychotherapy, Department of Psychology, Saarland University, Saarbruecken, Germany; ^2^MediClin Bliestal-Clinics, Blieskastel, Germany

**Keywords:** aggressive behavior, passive aggression, self-directed aggression, depression, test development

## Abstract

**Background:** To date, most research on aggression in mental disorders focused on active-aggressive behavior and found self-directed and other-directed active aggression to be a symptom and risk-factor of psychopathology. On the other hand, passive-aggressive behavior has been investigated less frequently and only in research on psychodynamic defense mechanisms, personality disorders, and dysfunctional self-control processes. This small number of studies primarily reflects a lack of a reliable and valid clinical assessment of passive-aggressive behavior. To address this gap, we developed the Test of Passive Aggression (TPA), a 24-item self-rating scale for the assessment of self-directed and other-directed passive-aggressive behavior.

**Method:** Study 1 examined the internal consistency and factorial validity of the TPA in an inpatient sample (*N* = 307). Study 2 investigated the retest-reliability, internal consistency, and construct validity (active aggression, personality traits, impulsivity) of the TPA in a student sample (*N* = 180).

**Results:** In line with our hypothesis, Exploratory Structural Equation Modeling revealed an acceptable to good fit of a bi-factorial structure of the TPA (*Chi-square-df-ratio* = 1.98; *RMSR* = 0.05, fit.off = 0.96). Both TPA scales showed good to excellent internal consistency (*α* = 0.83–0.90) and 4-week retest-reliability (*r*_*tt*_ = 0.86). Correlations with well-established aggression scales, measures of personality, and impulsivity support discriminant and convergent validity of the TPA.

**Conclusions:** The TPA is a reliable and valid instrument for the assessment of self-directed and other-directed passive-aggressive behavior.

## Introduction

Aggressive behavior is any behavior intended to harm oneself or others directly or indirectly (Buss, 1961). It can be differentiated into active and passive forms (Allen and Anderson, 2017). *Active* aggressive behavior comprises all forms of behavior including active engagement in the application of psychological or physical impairment, e.g., insulting someone or deliberate self-harm. *Passive* aggressive behavior is characterized by harmful inactivity and omission of active engagement, e.g., a lack of social support or neglect of one's own psychological needs. Therefore, aggression *per se* characterizes harmful behavior, but not to a personality trait (e.g., impulsivity), emotion (e.g., anger), or cognition (e.g., hostile attribution; Baron and Richardson, 2004). However, aggressive behavior tends to be stable over the life course and is thus assumed to represent a trait-like behavioral tendency (Huesmann et al., 2009). Although aggressive behavior is an evolutionary-based human problem-solving behavior (Buss and Shackelford, 1997), which can be adaptive in some contexts (Georgiev et al., 2013; Edmondson et al., 2016), it is also associated with high individual and societal costs (Laing and Bobic, 2002; Heilbron and Prinstein, 2008). Among other relevant risk factors for aggressive behavior [e.g., childhood sexual abuse (Fliege et al., 2009), hopelessness (Fox et al., 2015), impulsivity (Bresin, 2019)], aggressive behavior is related to psychopathology (Genovese et al., 2017). Prevalence rates of aggressive behavior in clinical samples exceed those of the general population, for both self-directed aggressive behavior [e.g., life-time prevalence of self-harm: 21 vs. 4% (Briere and Gil, 1998)] and other-directed aggressive behavior [e.g., 12 months prevalence of other-directed violence: 8–37% vs. 2% (Swanson et al., 2015)]. However, it is important to note that the vast majority of individuals affected by mental disorders does not show higher levels of aggressive behavior than the general population (Varshney et al., 2016). Nevertheless, individuals with mental disorders are an important population for research into aggressive behavior and for prevention of self-directed and other-directed aggressive behavior (Taft et al., 2012; Dutton and Karakanta, 2013; Hawton et al., 2013; Augsburger and Maercker, 2020).

Active aggressive behavior has been investigated extensively, which is reflected in the existence of well-established theoretical frameworks and many psychometric tests (Parrott and Giancola, 2007). For detailed information and reviews regarding active aggressive behavior in mental disorders, see, for e.g., Cafferky et al. (2018), Dutton and Karakanta (2013), Hawton et al. (2013), and Taft et al. (2012). By contrast, research on passive-aggressive behavior is relatively scarce, resulting in a smaller number of theories and a lack of psychometric tests (Parrott and Giancola, 2007). The term *passive aggression* was first used to characterize behavior of soldiers in World War II who acted in non-compliant ways to their superior's orders (Millon, 1993). In the aftermath, passive-aggressive personality disorder was included in the first version of the *Diagnostic and Statistical Manual of Mental Disorders* (*DSM*) and was characterized primarily by a set of behavioral symptoms (e.g., procrastination) (American Psychiatric Association, 1952). However, during various revisions of the *DSM*, the concept lost its substantive distinctiveness and was later renamed negativistic personality disorder. Thereby, affective symptom (e.g., moodiness) and cognitive characteristics (e.g., negativistic attitudes) were included and resulted in a large overlap with other personality disorders (Hopwood and Wright, 2012). This lack of clarity ultimately led to less research on passive-aggressive personality disorder and finally to its exclusion from *DSM-5* (American Psychiatric Association, 2013). Therefore, for a long time it was also difficult to capture the clinical significance of the concept, since associated characteristics (e.g., moodiness) were partly subject to the concept or not. Given that research into other psychological fields [e.g., organizational psychology (Baron and Neuman, 1996; Neuman and Baron, 1998)] demonstrated firmly the relevance of passive-aggressive behavior, it is also important to re-examine passive-aggressive behavior from a clinical perspective. Apart from nosological research into passive-aggressive personality disorder, mainly two theoretical approaches have inspired clinical research into passive-aggressive behavior: Research on *other-directed passive-aggressive behavior* mainly originated from psychodynamic research into *defense mechanisms* (Cramer, 2015), and research on s*elf-directed passive-aggressive behavior*—sometimes also referred to as *self-harm by omission* (Turp, 2007)—mainly focused on *self-control processes in depressive disorders* (Rehm, 1977).

### Passive-Aggression in Clinical Research

In psychodynamic research, *defense mechanisms* are defined as unconscious processes protecting the ego from emotional disturbance (e.g., fear) and instinctive urges (e.g., active aggression; Freud, 1936). Defense mechanisms are supposed to represent relatively stable traits (Bond, 2004), which are activated by internal or external conflicts (Segal et al., 2007). According to their capability to resolve conflicts, they are classified into mature, neurotic, and immature defenses (Andrews et al., 1993), with immature defense mechanisms being associated with childhood trauma or neglect (Romans et al., 1999; Nickel and Egle, 2006). Passive-aggressive behavior is conceptualized as an immature defense mechanism due to its negativistic and covert nature (Andrews et al., 1993; Schauenburg et al., 2007), thereby contributing to the suppression of emotional conflicts and impaired problem-solving capabilities (Cramer, 2015). Higher levels of passive-aggressive defense mechanisms are associated with more severe symptoms of anorexia nervosa (Tordjman et al., 1997), acute stress disorder (Santana et al., 2017), adjustment disorder (Ghazwin et al., 2017), borderline personality disorder (Zanarini et al., 2013), and deliberate self-harm (Baykara and Alban, 2018).

*The self-control theory of depression* is based on Kanfer's (1971) behavioral self-control model that proposes that individuals control their behavior using a feedback loop of *self-monitoring, self-evaluation, and self-reinforcement*. These processes are assumed to be distorted in patients with depressive symptoms due to dysfunctional cognitive biases and negative attributional styles (Rehm, 1977), which are supposed to develop in late childhood and get activated in stressful situations (Wang et al., 2010; Hu et al., 2015; Schierholz et al., 2016). The tendency of depressed patients to selectively focus on adverse stimuli and events (De Raedt and Koster, 2010) and to attribute these in a dysfunctional way (Hu et al., 2015) results in a negative self-evaluation (Orchard and Reynolds, 2018). Such a negative self-evaluation, in turn, is supposed to lead to excessive self-punishment and low levels of self-reward (Ciminero and Steingarten, 1978; Rozensky et al., 1981). Notably, the latter represents a form of self-directed passive-aggressive behavior and contributes to the development of depression (Fuchs and Rehm, 1977).

In summary, both approaches, in line with research on passive-aggressive personality disorder (Hopwood and Wright, 2012; Newton-Howes et al., 2015; Hopwood, 2018), assume that passive-aggressive behavior constitutes a relatively stable behavioral tendency that gets activated when individuals are exposed to internal or external stressors. Furthermore, passive-aggressive behavior is supposed to result from dysfunctional monitoring and evaluation processes, which are supposed to arise from negative childhood experiences. Therefore, passive aggression is assumed to be both a risk factor for and a result of psychopathology and interpersonal conflicts.

### Psychometric Tests on Passive-Aggressive Behavior

The availability of a valid psychometric test is important for the development of a research field. To give an example, resilience research is closely related to the development of the sense of coherence questionnaire (Antonovsky, 1987; Eriksson and Lindström, 2006). The development of psychometric tests needs to follow the assumptions of the classical or probabilistic test theory (Hambleton and Jones, 1993). Moreover, tests need to fulfill quality criteria (i.e., objectivity, reliability, and validity). Although psychometric tests originated from the above-mentioned research traditions show good overall psychometric properties, their suitability for assessing passive-aggressive behavior is limited by their item content (e.g., Fydrich et al., 1997; Kuhl and Kazén, 1997; Mezo and Short, 2012). To date, clinical tests (i.e., of passive-aggressive personality disorders, defense mechanisms, or self-control mechanisms) assess broader nosological categories (including cognitions, emotions, and personality traits) instead of passive-aggressive behavior only. Additionally, psychometric tests from other psychological fields assess passive-aggressive behavior in very specific contexts (i.e., at the workplace) or from victim instead of perpetrator perspective (e.g., regarding social ostracism), and are thus not applicable in the clinical context (Williams and Sommer, 1997; Neuman and Baron, 1998, 2005). Therefore, in order to facilitate clinical research into passive-aggressive behavior, the current research project aimed at developing and validating the *Test of Passive Aggression* (TPA), a behavior-based test for the assessment of both self-directed and other-directed passive aggression.

## Methods

### Scale and Item Construction

Development of the TPA followed guidelines for the development of psychometric tests according to the classical test theory (Hambleton and Jones, 1993). First, the authors (CS, ME, SKS, HM, and TM) agreed on the definition of self-directed and other-directed passive-aggressive behavior: Passive-aggressive behavior is a stable behavioral disposition to harm oneself or others by omission in reaction to internal or external stressors. The test was supposed to consist of one scale for the assessment of self-directed passive aggression (TPA-SD) and another scale for the assessment of other-directed passive-aggression (TPA-OD). Second, the first author (CS) created a set of items, all of which followed the following pattern: (internal or external stressor) + (unpleasant feeling) + (passive-aggressive behavioral reaction). The scenarios were chosen to fit different backgrounds and thus reflect a broad range of daily scenarios (e.g., an argument with a partner, conflicts at work, or personal failure) to be more inclusive than previous instruments that were limited to specific contexts (e.g., conflicts at work). Even though all items were formulated by the first author (CS), previous assessment of passive-aggressive defense mechanisms and passive-aggressive personality disorder served as a basis for item generation of the TPA-OD scale (e.g., Andrews et al., 1993; Kuhl and Kazén, 1997). Items of the TPA-SD scale followed the rationale of the self-control theory of depression and therefore focused on behavioral patterns which deny one-self rewards, reinforcement, or self-satisfaction (Rehm, 1977; Mezo and Short, 2012). To reflect the stable character of passive-aggressive behavior, a five-point scale ranging from “1 = *very unlikely*” to “5 = *very likely*” was used, asking the respondents to estimate the probability to react in the described manner in general. Initially, 16 items each for the assessment of self-directed and other-directed passive-aggression were developed. At this point, 32 items for the assessment of active aggression had been formulated but were discarded in the following steps due to expert ratings and test statistics (mean item severity <0.20). Third, a pilot study was conducted to provide primarily information on test statistics (i.e., item severity, inter-item correlation) in a sample of adult (age ≥ 18 years) psychology students [*N* = 102, 86.27% females, *M*(age) = 21.44 years, *SD*(age) = 3.29]. For additional information on the pilot data, see Supplementary Material A. Based on these results, the authors (CS, EM, SS, HM, TM) refined the items and increased their number to 18 per scale (see Table 1 for the 36-item version of the TPA). Fourth, we analyzed the factorial structure in a larger clinical sample (Study 1), and fifth, we examined retest-reliability and construct validity of the TPA (Study 2).

**Table 1:** Items of the 36-item version of the TPA.

**Item**	**Item content**	**Scale**
1	If I am feeling down, I do not allow myself to do things or activities that would have actually been good for me	TPA-SD
2	If I am successful at something, I overthink that success until I find something bad about it	TPA-SD
3	If I want to teach someone a lesson, I do not respond to his/her contact attempts and ignore his/her messages	TPA-OD
4	If I performed well at work/at study, I avoid talking about it with my family, because I do not think I really deserve any commends on it	TPA-SD
5	If someone has hurt my feelings, I refuse to support that person in difficult situations	TPA-OD
6	When I am irritated and notice that a friend is sad or upset, I do not ask what is bothering him/her	TPA-OD
7	If I have information that is useful to a person, I do not like, I still forward the information to that person^*^	TPA-OD
8	If I receive a present from someone, I will accept it, even when I have the feeling that I do not really deserve it^*^	TPA-SD
9	If I feel I have upset someone close to me, I subsequently forgo planned enjoyable activities, such as a good meal or wellness	TPA-SD
10	When someone takes time to support me in a stressful situation, I resist accepting the help because I cannot imagine truly deserving this support	TPA-SD
11	If I could help a person I do not like with a problem, I refuse from doing it	TPA-OD
12	When I plan my free time, I do not do what I assume is enjoyable for me, but rather I go by what other people, such as friends or family members, want me to do	TPA-SD
13	If I am angry at someone, I ignore that person and their needs	TPA-OD
14	If I am feeling down, I throw myself into taking care of everyday responsibilities, for e.g., work or housekeeping, instead of consciously doing something good for myself	TPA-SD
15	If I fail at something, I cancel planned leisure activities, for e.g., going to the cinema or going for shopping	TPA-SD
16	If I feel the need for interpersonal affection or an uplifting activity, I still continue in my daily routine instead of fulfilling that need	TPA-SD
17	If I have made a mistake, I refuse emotional support from other people afterwards	TPA-SD
18	If I am sad, I refuse to participate in activities that could cheer me up	TPA-SD
19	If someone at work/at university annoys me, I reduce my involvement in our teamwork	TPA-OD
20	If I am dissatisfied with someone's behavior, I do not address him/her directly, but react coolly or disinterestedly to his/her behavior	TPA-OD
21	If I have resources, for e.g., money or time, at my free disposal, I use them to make myself feel good^*^	TPA-SD
22	If I am angry at someone, I will not provide that person with emotional support	TPA-OD
23	When someone important to me has hurt me emotionally, I suspend existing habits with him/her, such as going for walks together or talking on the phone regularly	TPA-OD
24	If I am dissatisfied with the commitment of my colleagues in a team effort, I start performing at the minimum level required afterwards	TPA-OD
25	If someone has denied helping me with a problem, I am still willing to help that person with similar problems^*^	TPA-OD
26	If I know that someone I am upset with is about to make a mistake I do not make him/her aware of it	TPA-OD
27	If my partner does not see my needs, I pay him/her back by, for e.g., doing the shopping or cooking just for me	TPA-OD
28	Even when I am feeling bad, I still care more about other people's needs than my own	TPA-SD
29	If I am upset with a friend, I exclude him/her from enjoyable activities, for e.g., from trips or going to the cinema	TPA-OD
30	Even though I've had a particular wish for years, such as going on vacation or trying a new leisure activity, I always miss out on opportunities to fulfill that wish	TPA-SD
31	After an argument with someone close to me, I still try to give the day a positive turn afterwards, for e.g., by engaging in a hobby or some other pleasant leisure activity^*^	TPA-SD
32	When I doubt whether I have done a task well, I refuse to be complimented or rewarded by other people for this accomplishment	TPA-SD
33	After an argument with my partner, I refuse to show him/her any tenderness	TPA-OD
34	If I am upset with someone at work/at study, I do not give that person praise that they would have actually deserved	TPA-OD
35	When someone gives a kind comment to me, I am convinced that person just wants to be nice	TPA-SD
36	If a friend has disappointed me, I wait until he/she makes the first step toward me before getting back to him/her	TPA-OD

*Both studies used German versions of the Test of Passive Aggression. SD, self-directed aggression; OD, other-directed aggression*.

* *inverse formulation*.

## Study 1

Study 1 aimed at initially validating and refining the 36-item version of the TPA. For this purpose, it examined the assumed bi-factorial structure of the TPA as well as its internal consistency and the relationships between other-directed and self-directed passive-aggression and depressive symptoms, somatoform symptoms, anxiety, and global psychopathological symptom severity.

### Materials and Methods of Study 1

#### Participants and Procedure

A total of 319 patients 18 years and older participated in Study 1. Patients were recruited at a German psychosomatic clinic (MediClin Bliestal-Clinics, Blieskastel), where they received a 5-to-6-week multidisciplinary inpatient treatment. Twelve patients were excluded due to more than four missing values on the TPA. For the characteristics of the final sample see Table 2. Participants gave written informed consent according to the Declaration of Helsinki (World Medical Association, 2013) and completed the assessments described below on their arrival (M1) and again at their discharge (M2). Study 1 was preregistered at the German Clinical Trials Register (www.drks.de, ID: DRKS00014002), an online platform for preregistration of clinical studies.

**Table 2:** Descriptive sample characteristics Study 1.

	**M1**	**M2**
Female	70.7%	68.0%
Age mean	53.21 years	53.25 years
Age SD	7.86	7.68
Age range	20–74 years	20–72 years
Adjustment disorders	39.60%	40.80%
Mood disorders	29.35%	31.61%
Somatoform disorders	17.41%	15.52%
Anxiety disorders	9.56%	10.35%
Comorbidity rate	48.50%	46.10%

*Total N_M1_ = 307; Total N_M2_ = 180. SD, Standard Deviation. Psychological diagnoses according to ICD-10. Comorbidity rate indicates the rate of at least one comorbid diagnosis*.

#### Measures

The 36-item version of the TPA was used as a self-report assessment of passive-aggressive behavior. This TPA version consisted of 18 items for the assessment of self-directed passive-aggressive behavior and 18 items for the assessment of other-directed passive-aggressive behavior. Therefore, a bi-factorial structure—with one factor representing self-directed passive-aggressive behavior and the other factor representing other-directed passive-aggressive behavior—was assumed.

The Beck Depression Inventory–II (BDI-II) assessed the severity of depressive symptoms according to the *Diagnostic and Statistical Manual of Mental Disorders-IV* (*DSM-IV*; American Psychiatric Association, 1994; Beck et al., 1996; Hautzinger et al., 2009). Each of the 21 items is rated on a 0 to 3 scale, with higher scores indicating more severe depressive symptoms. The BDI-II is a well-established measure of depression with acceptable to excellent retest-reliability (*r*_*tt*_ = 0.73 to 0.96; Wang and Gorenstein, 2013) and high validity (Hautzinger et al., 2009).

The Beck Anxiety Inventory (BAI) is a screening instrument to assess anxiety symptoms (Beck and Steer, 1993; Margraf and Ehlers, 2002). It consists of 21 items, rated on a 0 to 3 scale, with higher scores indicating more severe levels of anxiety. Whereas, the BAI shows excellent internal consistency (α = 0.91) and high validity, its retest-reliability is acceptable (*r*_*tt*_ = 0.78; Geissner and Huetteroth, 2018).

The Hamburg Modules for the Assessment of Psychosocial Health in Clinical Practice (HEALTH-49; Rabung et al., 2007) assesses nine mental health-related subscales [somatoform complaints (SOM), depressiveness (DEP), phobic anxiety (PHO), psychological well-being, interactional problems, self-efficacy, activity and participation, social support, and social stress]. The scores of SOM (*α* = 0.82), DEP (*α* = 0.88), and PHO (*α* = 0.82–86) can be combined to a global symptom severity index (GSI, *α* = 0.89; Rabung et al., 2009).

#### Statistical Analysis

Exploratory Structural Equation Modeling (ESEM) was performed using *R* (Gascon et al., 2013) and the *psych* package (Revelle, 2015). All remaining statistical analyses were conducted using IBM SPSS Statistics version 25 (IBM Corp, 2017).

##### Item Reduction

As also indicated in the preregistration, Study 1 aimed to reduce items per scale to 12 for optimizing scales economy for use in clinical settings. For both subscales, item reduction followed a three-step procedure. First, all items with an item difficulty below 0.20 and above 0.80 were eliminated. Items severity was calculated by dividing the mean value per item by the maximum value per item (i.e., lower values represent higher item difficulty). Second, all items with an inter-item correlation below 0.30 were removed. Third, a primary axis factoring was conducted. The primary axis factoring was used for item reduction in order to choose the most representing items of each scale (i.e., items with the highest loading on the one factor solution). Subsequently, the bi-factorial structure of the refined TPA was analyzed using ESEM (see below).

##### Factorial Validity

The model-fit of the refined bi-factorial solution was analyzed using ESEM, which combines exploratory factor analysis with the assessment of model fit using structural equation modeling (SEM; Revelle, 2015). ESEM was found to be more appropriate than SEM for analyzing psychological instruments (Marsh et al., 2010, 2014). With respect to the current study, it is of particular importance that ESEM does not require zero cross-loadings, as other-directed and self-directed aggressive behavior are known to be strongly related (O'Donnell et al., 2015). All analyses used minimum residual estimations with pairwise exclusion and oblimin rotation. As the primary measures provided by the *psych* package, Chi-square-df-ratio (good fit < 2), Root Mean Square of the Residuals (RMSR, good fit > 0.05), and fit based upon off diagonal values (fit.off, good fit > 0.95) were used as fit indices (Hu and Bentler, 1999). Models were estimated for M1 and M2.

##### Internal Consistency and Item-Total Correlation

Internal consistency was calculated using Cronbach's alpha (*α*; Cronbach, 1951) and McDonald's omega (ω; McDonald, 1999). According to Mallery and George (2003), *internal consistencies* were interpreted as follows: >0.90 = excellent; >0.80 = good; >0.70 = acceptable; >0.60 = questionable; >0.50 = poor; and <0.50 = inacceptable. Internal consistencies as well as item-total correlations were analyzed for both points of assessment.

##### Association Between Passive-Aggressive Behavior and Symptom Clusters

Bivariate associations between passive-aggressive behavior and psychopathological symptom severity (BDI-II, BAI, SOM, DEP, PHO, and GSI) were analyzed using Pearson correlations. Given the strong correlation between depressive symptoms, somatoform symptoms, and anxiety symptoms (Rabung et al., 2009), two multiple regression analyses were conducted to assess the incremental proportion of variance of passive-aggressive behavior explained by each symptom domain (SOM, DEP, and PHO), under mutual control for the other symptom domains. Thereby, we aimed at investigating the unique association between depressive symptoms and passive-aggressive behavior irrespective of general psychopathology.

### Results of Study 1

#### Item Reduction

One item of the TPA-OD was removed due to an item difficulty below 0.20 (item 27). Another five items were removed since all inter-item correlations fell below 0.30 (items 6, 7, 24, 25, 26). All remaining items demonstrated factor loadings above 0.30 on the one-factor solution of a principal axis factoring. All items of the TPA-SD showed an item difficulty above 0.20 and below 0.80. Four items were removed since all inter-item correlations were below 0.30 (items 1, 8, 21, 31). All remaining items showed factor loadings above 0.30 on the one-factor solution of a primary axis factoring. In order to reduce the number of items to 12, the two items exhibiting the lowest factor loading were removed (items 4, 28).

#### Model-Fit of the Two-Factor Solution

Fit indices of both measure points are presented in Table 3. Overall, ESEM revealed an acceptable to good model fit for the bi-factor solution of the refined TPA. Factor loadings of all items on the respective factor ranged between 0.45 and 0.69 at M1 and between 0.38 and 0.77 at M2. The correlation between TPA-SD and TPA-OD was strong at M1 (*r* = 0.52) and medium at M2 (*r* = 0.38).

**Table 3:** Model fit of ESEM.

	**RMSR**	**Fit.off**	**Chi-square**	**Chi-square-df-ratio**
M1	0.05	0.96	452.83	1.98
M2	0.06	0.96	372.09	2.46

*RMSR, Root Mean Square of the Residuals; Fit.off, Fit based upon of diagonal values*.

#### Internal Consistency and Item-Total Correlation

Internal consistencies were good at M1 (*α*_other−directed_ = 0.83; ω _other−directed_ = 0.83; α_self−directed_ = 0.84; ω _other−directed_ = 0.85) and M2 (*α*_other−directed_ = 0.86; ω _other−directed_ = 0.86; *α*_self−directed_ = 0.89; ω _self−directed_ = 0.89). Item-total correlations at M1 ranged between 0.40 and 0.61 for the TPA-OD and the TPA-SD. Similarly, at M2 item-total correlations ranged from 0.36 to 0.66 for the TPA-OD and from 0.38 to 0.73 for the TPA-SD. Further item characteristics are presented as Supplementary Material B.

#### Association Between Passive-Aggressive Behavior and Symptom Clusters

Bivariate correlations between both TPA scales and psychopathological symptom levels are presented in Table 4. In line with our hypotheses, both self-directed and other-directed passive-aggressive behavior were associated with all symptom domains. However, an association of other-directed passive-aggressive behavior with anxiety levels was represented in the BAI only. Results of the multiple regression analyses for the prediction of self-directed and other-directed passive-aggressive behavior based on depressive, phobic, and somatic symptoms are presented in Tables 5, 6. In line with self-control theory of depression, self-directed passive-aggressive behavior demonstrated a particularly strong unique association with depressive symptoms. By contrast, for none of the symptom domains a unique association with other-directed passive-aggressive behavior was detected.

**Table 4:** Bivariate correlations of both TPA scales and symptom severities.

	**TPA-OD**	**TPA-SD**
HEALTH-49-GSI	0.19^*^	0.39^**^
HEALTH-49-DEP	0.15^*^	0.39^**^
HEALTH-49-SOM	0.14^*^	0.34^**^
HEALTH-49-PHO	0.17^*^	0.26^**^
BDI-II	0.14^*^	0.40^**^
BAI	0.09	0.39^**^

*HEALTH-49, The Hamburg Modules for the Assessment of Psychosocial Health in Clinical Practice; GSI, general symptom index; SOM, somatoform complaints; DEP, depressiveness, PHO, phobic anxiety; BDI-II, Beck-Depression Inventory–II; BAI, Beck-Anxiety Inventory*.

* *p < 0.05*;

** *p < 0.001*.

**Table 5:** Multiple regression for prediction of TPA-SD.

	**Beta**	**T**	***p***
HEALTH-49-DEP	0.27	3.79	<0.001
HEALTH-49-SOM	0.17	2.46	0.014
HEALTH-49-PHO	0.05	0.41	0.679

*Criterium is the self-directed passive-aggression scale of the TPA. HEALTH-49, The Hamburg Modules for the Assessment of Psychosocial Health in Clinical Practice; GSI, general symptom index; SOM, somatoform complaints; DEP, depressiveness, PHO, phobic anxiety. Model: F_(3,280)_ = 19.01; R^2^ = 0.17; p < 0.001*.

**Table 6:** Multiple regression for prediction of TPA-OD.

	**Beta**	**T**	***p***
HEALTH-49-DEP	0.06	0.78	0.439
HEALTH-49-SOM	0.05	0.70	0.485
HEALTH-49-PHO	0.12	1.68	0.094

*Criterium is the other-directed passive-aggression scale of the TPA. HEALTH-49, The Hamburg Modules for the Assessment of Psychosocial Health in Clinical Practice; GSI, general symptom index; SOM, somatoform complaints; DEP, depressiveness; PHO, phobic anxiety. Model: F _(3,280)_ = 3.44; R^2^ = 0.04; p = 0.017*.

### Discussion of Study 1

In Study 1, the 36-items version of the TPA was evaluated and reduced to its final 24-items form. Applying ESEM, we confirmed the bi-factorial structure of the TPA, consisting of TPA-SD and TPA-OD. Additionally, Study 1 verified good internal consistencies for both scales.

Study 1 also revealed small to moderate associations between passive-aggressive behavior and psychopathological symptom severity in a clinical inpatient sample. Other-directed passive-aggression was associated with global psychopathological symptom severity, but not with specific symptom domains. These results are in line with previous research showing that active other-directed aggressive behavior (Genovese et al., 2017) as well as passive-aggressive personality (Laverdière et al., 2019) and passive-aggressive defense style (Bond, 2004) are relevant in a broad range of mental disorders. Thus, other-directed passive-aggressive behavior might represent a reaction to general mental distress. This view also contributed to the removal of passive-aggressive personality disorder from *DSM-5* (Wetzler and Jose, 2012).

By contrast, in addition to its association with global psychopathological symptom burden, self-directed passive-aggressive behavior exhibited specific unique associations with depressive and somatoform symptoms. In bivariate and multivariate analyses, depressive symptoms were the numerically strongest correlates for self-directed passive-aggressive behavior. The association of self-directed passive-aggressive behavior and depressive symptoms is in line with the self-control theory postulating that dysfunctional self-monitoring and self-evaluation processes in depressed patients lead to a lack of self-reinforcing behavior (i.e., self-harm by omission; Fuchs and Rehm, 1977; Rehm, 1977; Rozensky et al., 1977; Roth and Rehm, 1980). Furthermore, this association corresponds to previous research on active aggressive behavior indicating that both forms of self-directed aggressive behavior are strongly related to depression severity (Hawton et al., 2013; Plener et al., 2015; Harford et al., 2018).

Like depressive symptoms, somatoform symptoms explained an incremental proportion of variance in self-directed passive-aggressive behavior. Correspondingly, former research found high rates of comorbidity between somatoform disorders and passive-aggressive personality disorder (Bass and Murphy, 1995; Noyes et al., 2001). Since associations between somatization and self-directed passive-aggressive behavior have not yet been examined in previous research, our result needs to be replicated in further studies.

### Limitations

The following limitations have to be taken into account: In contrast to internal consistency, methods of Study 1 did not allow a valid examination of retest-reliability. The analysis of retest-reliability would have required a time interval, in which the concept of interest is supposed to be relatively stable. However, patients of Study 1 received a multidisciplinary intervention, including psychotherapeutic and psychopharmacological treatment, and therefore interventions sufficient to change levels of aggressive behavior (Jones R. M. et al., 2011; Karakurt et al., 2016; Kothgassner et al., 2020). Thus, there is the need of another study to further examine the retest-reliability of the TPA in a non-clinical context.

Given that, an additional assessment of other constructs (e.g., active aggression, impulsivity) would have exceeded capacities of clinical staff, we were also not able to assess construct validity in Study 1.

Another limitation refers to the diagnostic process in Study 1. Whereas, symptom severity was assessed using standardized measures (Beck and Steer, 1993; Beck et al., 1996; Rabung et al., 2007), psychiatric diagnoses were based on unstructured clinical interviews known to be less accurate than structured interviews (Miller et al., 2001). Therefore, our main analyses regarding associations between self-reported passive-aggressive behavior and symptom clusters were based on regression models relying on standardized measures instead of group comparisons. Future studies in clinical samples should use structured clinical interviews to enable valid analyses of group differences of passive-aggressive behavior.

## Study 2

Study 1 resulted in the final 24-item version of the TPA and demonstrated its factorial validity and internal consistency. However, data on test-retest reliability and construct validity were still missing. Therefore, Study 2 aimed at closing this gap.

### Materials and Methods of Study 2

#### Participants and Procedure

Participants of Study 2 were adult (age ≥ 18 years) undergraduate students, recruited in psychology lectures at Saarland University. Participants received course credits for their participation. After given written informed consent in line with the Declaration of Helsinki (World Medical Association, 2013), data was collected using the online survey platform *SoSci Survey* (Leiner, 2014). To assess the test-retest-reliability of the TPA, participants received survey links at three 14-day intervals via email. Additionally, participants completed German versions of the *Short Questionnaire for the Assessment of Components of Aggression* [K-FAF, Heubrock and Petermann (2008)], the *NEO Five Factor Inventory* [NEO-FFI, Borkenau and Ostendorf (1994), Costa and McCrae (1989)], and the short version of the *Barratt Impulsiveness Scale* [BIS-15, Meule et al. (2011)] at the first point of assessment (M1). See Table 7 for sample characteristics. Study 2 was also preregistered at the German Clinical Trials Register (www.drks.de, ID: DRKS00014607).

**Table 7:** Descriptive sample characteristics Study 2.

	**M1**	**M2**	**M3**
N	180	140	133
Women	75.6%	77.1%	77.4%
Age mean^a^	21.55 years	21.64 years	21.53 years
Interval to M1 mean	–	12.44 days	26.90 days
Interval to M1 range	–	7–20 days	21–34 days

a *Age range for all measure points was 18–32 years*.

#### Measures

Self-directed and other-directed passive-aggressive behavior were assessed using the 24-item version of the TPA (see Study 1).

The K-FAF includes 49 items assessing aggression on five dimensions (spontaneous aggression, reactive aggression, irritability, auto-aggression, aggression inhibition; Heubrock and Petermann, 2008). The K-FAF was chosen because its scales allow for an economic assessment of self- and other-directed aggression. However, the internal consistencies of the K-FAF scales are only poor (aggression inhibition; *α* = 0.55) to good (irritability; *α* = 0.84). We hypothesized that convergent validity of the TPA-OD should be reflected in at least medium-sized relationships between the TPA-OD and the active aggression scales of the K-FAF (i.e., spontaneous and reactive aggression). Self-directed passive-aggressive behavior is supposed to result from negative self-monitoring and self-evaluation processes in depression (Rehm, 1977). Therefore, it should be closely related to self-conscious emotions (Laye-Gindhu and Schonert-Reichl, 2005), self-reproach (Jinting and Hairong, 2019), and self-criticism (Gilbert et al., 2010). The auto-aggression scale of the K-FAF comprises these aspects. Thus, we expected the TPA-SD and the auto-aggression scale to show an at least medium-sized correlation.

The NEO-FFI is a short version of the *Revised NEO Personality Inventory* (Costa and Mac Crae, 1992) and assesses five personality traits (openness, conscientiousness, extraversion, agreeableness, neuroticism) using 60 items (Costa and McCrae, 1989; Borkenau and Ostendorf, 1994). The NEO-FFI is a well-established and widely used instrument in research on the relationship between personality traits and aggressive behavior (Burton et al., 2007; Grumm and von Collani, 2009; Carvalho and Nobre, 2019). The internal consistencies of its scales are questionable (openness; *α* = 0.61 to 0.71) to good (neuroticism, *α* =0.81 to 0.85). Given that other-directed passive-aggressive behavior has been shown to be associated with interpersonal conflicts (Laverdière et al., 2019), convergent validity of the TPA-OD would be reflected in an at least medium-sized negative correlation with agreeableness (Jones S. E. et al., 2011). Neuroticism is related to lower levels of internal control, self-esteem, and general self-efficacy (Judge et al., 2002). Thereby, it reflects one facet of self-evaluation (Chang et al., 2012). Thus, we expected an at least medium-sized relationship between neuroticism and TPA-SD to evidence convergent validity (Brown, 2009). Since conscientiousness is closely related to delayed gratification (a construct that needs to be distinguished from self-directed passive-aggression; Furnham and Cheng, 2019), an at the most small correlation between conscientiousness and TPA-SD should reflect its discriminant validity.

The BIS-15 is a short version of the Barratt Impulsiveness Scale−11 (Patton et al., 1995; Preuss et al., 2008), the standard assessment of impulsivity (Stanford et al., 2009). Its economic 15-item version showed good reliability (*α* = 0.81; Meule et al., 2011). Impulsivity, a predisposition for rash and spontaneous behavior, is a strong predictor for active other-directed (Bresin, 2019) and self-directed (Gvion and Apter, 2011; Hamza et al., 2015) aggressive behavior. By contrast, passive-aggressive behavior is supposed to harm by omission and should thus be explicitly characterized by a lack of impulsive action (Buss, 1961; Parrott and Giancola, 2007). However, given passive-aggressive behavior is also positively associated with active aggressive behavior, we expected passive-aggressive behavior to be independent from impulsiveness, reflected in an at the most small correlation between the TPA scales and the BIS-15 as further support of discriminant validity.

#### Data Analysis

All analyses were performed using IBM SPSS Statistics 25 (IBM Corp, 2017). For the evaluation of test-retest-reliability, Pearson correlations for both TPA scales were calculated for all points of assessment. Internal consistencies were analyzed for all measure points using α (Cronbach, 1951) and ω (McDonald, 1999). As in Study 1, internal consistencies were interpreted as follows: >0.90 = excellent; >0.80 = good; >0.70 = acceptable; >0.60 = questionable; >0.50 = poor; and <0.50 = inacceptable (Mallery and George, 2003). To evaluate construct validity Pearson correlations were calculated for both TPA scales with the BIS-15 and the subscales of the K-FAF and the NEO-FFI.

### Results of Study 2

#### Reliability

For both TPA scales retest-reliabilities were good to excellent (see Table 8). The internal consistencies of TPA-OD were good and the TPA-SD showed good to excellent internal consistencies (see Table 9).

**Table 8:** Retest-reliability of the TPA scales.

	**M1**	**M2**	**M3**
M1	–	0.86^a^	0.86^c^
M2	0.84^b^	–	0.91^e^
M3	0.86^c^	0.90^d^	–

*Correlations above the diagonal are retest-reliabilities for the self-directed passive-aggressive behavior scale. Correlations beneath the diagonal are retest-reliabilities for the other-directed passive-aggressive behavior scale*.

a *n = 140*,

b *n = 139*,

c *n = 133*,

d *n = 120*,

e *n = 119*.

**Table 9:** Internal consistencies of the TPA scales.

	**M1**		**M2**		**M3**	
	***α***	***ω***	***α***	***ω***	***α***	***ω***
Self-directed	0.85^a^	0.85^a^	0.87^b^	0.87^b^	0.90^c^	0.90^c^
Other-directed	0.84^a^	0.84^a^	0.87^b^	0.89^b^	0.89^c^	0.89^c^

*Coefficients are internal consistencies for each measure point*.

a *n = 180*,

b *n = 139*,

c *n = 133*.

#### Validity

Bivariate correlations between the TPA scales, the BIS-15, the subscales of the NEO-FFI, and the K-FAF are presented in Table 10. Construct validity of the TPA-OD was supported by significant medium to large correlations with spontaneous aggression and reactive aggression as well as a negative medium correlation with agreeableness. Discriminant validity of the TPA-OD was confirmed by a non-significant association with impulsiveness. Furthermore, construct validity of the TPA-SD was supported by large significant correlations with auto-aggression and neuroticism. Moreover, discriminant validity of the TPA-SD was confirmed by non-significant associations with impulsiveness and conscientiousness.

**Table 10:** Construct validity of the TPA scales.

	**Other-Directed**	**Self-Directed**
**K-FAF**		
Spontaneous aggression	0.42^**^	0.22^**^
Reactive aggression	0.54^**^	0.21^**^
Irritability	0.38^**^	0.21^**^
Auto-Aggression	0.27^**^	0.66^**^
Aggression inhibition	−0.10	0.16^*^
**NEO-FFI**		
Openness	−0.11	0.07
Conscientiousness	0.03	0.13
Extraversion	−0.23^**^	−0.47^**^
Agreeableness	−0.43^**^	−0.14
Neuroticism	0.25^**^	0.58^**^
**BIS-15**	0.13	0.03

*K-FAF, Kurzfragebogen zur Erfassung von Aggressivitätsfaktoren (short questionnaire for the assessment of components of aggression); NEO-FFI, NEO Five Factor Inventory; BIS-15, Barratt Impulsiveness Scale–Short Version*.

* *p < 0.05*,

** *p < 0.01*.

### Discussion of Study 2

Study 2 revealed high correlations between passive-aggressive behavior and active aggression, neuroticism, and agreeableness (inverse) as well as small insignificant correlations between passive-aggressive behavior and impulsivity and consciousness, thereby demonstrating convergent and discriminant validity of both the TPA-OD and TPA-SD scale.

As aggression is supposed to represent a trait-like behavioral tendency, which should remain stable over time (Huesmann et al., 2009), substantial retest-reliability is of critical relevance for its assessment. Both the TPA-OD and the TPA-SD scale showed good to excellent retest-reliabilities over a period of ~4 weeks. Moreover, both TPA scales showed good to excellent internal consistencies. Compared to well-established scales assessing active aggressive behavior, the TPA exhibits comparable or even higher reliability (see Table 11).

**Table 11:** Examples for retest-reliabilities and internal consistencies of aggression questionnaires.

	**r_***tt***_ (interval)**	***α***
TPA other-directed	0.86 (26.90 days)	0.83–0.89
TPA self-directed	0.86 (26.90 days)	0.84–0.90
AQ (Herzberg, 2003)	0.69–0.81 (31 days)	0.65–0.88
STAXI-2 AX-O (Rohrmann et al., 2013)	0.81 (6 weeks)	0.86
STAXI-2 AX-I (Rohrmann et al., 2013)	0.80 (6 weeks)	0.81
K-FAF spontaneous aggression (Heubrock and Petermann, 2008)	–	0.77
K-FAF reactive aggression (Heubrock and Petermann, 2008)	–	0.77
K-FAF auto-aggression (Heubrock and Petermann, 2008)	–	0.82
DSHI (Gratz, 2001)	0.68^a^ (3.3 weeks)	0.82
SIQ (Santa Mina et al., 2006)	0.88 (2 weeks)	0.83

*AQ, Buss and Perry Aggression Questionnaire; STAXi-2, State-Trait-Anger-Expression Inventory-2; K-FAF, Kurzfragebogen zur Erfassung von Aggressivitätsfaktoren (short questionnaire for the assessment of components of aggression); DSHI, Deliberate Self-Harm Inventory; SIQ, Self-Injury Questionnaire*;

a *Φ*.

Convergent validity of the TPA was supported by medium to high correlations between the corresponding TPA and K-FAF scales, which are well-established assessments of aggressive behavior. These associations underline the notion that passive-aggressive behavior represents a form of aggressive behavior, even though it is characterized by the absence of active behavioral engagement. Furthermore, in contrast to active aggressive behavior (Bresin, 2019), the TPA scales were not significantly correlated with impulsivity. This result provides further evidence for an increment of the assessment of passive-aggressive behavior above the general assessment of aggressive behavior.

According to the *DSM-IV*, individuals with passive-aggressive personality disorder are supposed to let others down, provide less social support, and be not trustworthy (American Psychiatric Association, 1994). These behavioral tendencies are assumed to lay the foundation for high rates of interpersonal conflicts (McCann, 1988). Therefore, the negative association between TPA-OD and agreeableness is in line with theoretical considerations. The same applies to the relationship between TPA-SD and neuroticism that supports the notion that self-directed passive-aggressive behavior might be driven by dysfunctional self-evaluation (Rehm, 1977; Chang et al., 2012). A lack of self-reinforcement could not only represent a form of self-directed aggressive behavior but also the capability for delayed gratification, which is associated with consciousness (Furnham and Cheng, 2019). Therefore, given that it is essential to aggressive behavior that it is committed with intention (Allen and Anderson, 2017), our finding of a small and insignificant association between the TPA-SD and consciousness supports the discriminant validity of the scale.

### Limitations

In contrast to Study 1, Study 2 allowed for the analysis of test-retest reliability and construct validity of the TPA by the use of a student sample. However, findings on the transferability of results from student samples to clinical samples or the general population are mixed (Henry, 2008; Boals et al., 2020). Therefore, future studies should examine the test-retest-reliability and construct validity of the TPA in a waiting list sample.

## General Discussion

Study 1 and Study 2 showed that the TPA is a reliable and valid assessment of other-directed and self-directed passive-aggressive behavior. In contrast to previous scales for the assessment of other-directed passive-aggressive behavior that were mostly targeting broader nosological categories, e.g., passive-aggressive personality disorder, the TPA-OD scale does not assess personality traits (e.g., hostility) or emotions (e.g., feelings of anger) but passive-aggressive behavior directly. This is a major advantage for investigating precursors and consequences of other-directed passive-aggressive behavior because it helps to minimize confounders. For example, when investigating the association between psychopathological symptoms and aggressive behavior, emotional confounders like anger or sadness are particularly relevant. Therefore, future studies should make use of this advantage of the TPA and investigate which (internal or external) variables predict other-directed passive-aggressive behavior and to what extent other-directed passive-aggressive behavior has a predictive value for the development and course of mental disorders.

Moreover, in contrast to previous assessments of passive-aggressive behavior, the TPA is the first to include an assessment of self-directed passive-aggressive behavior, which may be of major relevance in the context of depressive disorders (Rehm, 1977). The unique association of self-directed passive-aggressive behavior with depressive symptoms in Study 1 provided first evidence for this notion. Future clinical studies should investigate the longitudinal relationship between self-directed passive-aggressive behavior and depressive symptoms. These studies should also use structured clinical interviews to examine if self-directed passive-aggressive behavior occurs more frequently in depressive disorders than in other mental disorders.

As outlined in the introduction, passive-aggressive behavior is one dimension of the broader construct of aggressive behavior. Therefore, one might expect that many of the general assumptions on aggression also apply to passive-aggressive behavior (e.g., its emotional and cognitive precursors or its stability over the life course). Nevertheless, future studies need to test this hypothesis by investigating which personal and/or situational factors contribute to different expressions of aggressive behavior. In this context, Study 2 provided first insight by demonstrating passive-aggressive behavior to be independent from impulsivity.

## Conclusion

The TPA is a reliable and valid self-report instrument for the assessment of other-directed and self-directed passive-aggressive behavior. The current study indicates a substantial overlap between passive- and active-aggressive behavior, but it indicates that passive-aggressive behavior occurs independently from impulsiveness. Self-directed passive-aggressive behavior is significantly associated with depressive and somatoform symptoms. Future studies should assess both active- and passive-aggressive behavior in clinical samples to provide further insights into the relationships between aggressive behavior, intra- and interpersonal conflicts, and psychopathological symptoms.

## Data Availability Statement

The raw data supporting the conclusions of this article will be made available by the authors, without undue reservation.

## Ethics Statement

The studies involving human participants were reviewed and approved by the ethics comitee of the Saarland University. The patients/participants provided their written informed consent to participate in this study.

## Author Contributions

CS designed the study, organized sample recruitment, analyzed and interpreted the data, drafted the article, and prepared the final manuscript. MK helped to design the study and contributed to sample recruitment. TM, ME, SS, and HM contributed to conception and design of the study, supported the interpretation of the data, and commented on manuscript drafts. All authors read and approved the final manuscript.

## Conflict of Interest

The authors declare that the research was conducted in the absence of any commercial or financial relationships that could be construed as a potential conflict of interest.

## Supplementary Material

Data Sheet 1 Supplementary Material A and B.
