# Supplementary material for: Development and Psychometric Properties of the Test of Passive Aggression
Source: Front Psychol. 2021 Apr 26;12:579183. doi: 10.3389/fpsyg.2021.579183 (PMC8107391; doi:10.3389/fpsyg.2021.579183)
Supplement: Data Sheet 1 — Supplementary Material A and B. [file Data_Sheet_1.PDF]

Supplements: Development and psychometric properties of the Test of Passive Aggression

Schanz, C. G.[1], Equit M.[1], Schäfer, S. K.[1], Käfer, M. [2], Mattheus, H. K.[1], Michael, T.[1*]

[1] Clinical Psychology and Psychotherapy, Department of Psychology, Saarland University, Saarbruecken, Germany

[2] MediClin Bliestal-Clinics, Blieskastel, Germany

Supplement A. Pilot data

The pilot study was conducted in a sample of psychology students 18 years and older [*N* = 102, female = 86.27%; *M*(age) = 21.44 years, *SD*(age) = 3.29]. The pilot study aimed to allow for initial analyses of item quality and subsequent modifications of item content. Procedures for item reduction and factor analyses followed procedures described for Study 1 in the main manuscript. Table S1 shows English translations of the German items of the 32-item pilot version of the Test of Passive Aggression.

Table S1. *Items of the 32-item pilot version of the Test of Passive Aggression*

Item	Item content	Scale
1	If I fail at something, I hardly eat anything afterwards because I do not deserve it.	TPA-SD
2	If I am disappointed by someone, I show that person my disapproval by not reaching out for him/her again.	TPA-OD
3	When we do things together, I don't say what I want so no one has to take consideration into me.	TPA-SD
4	If I am angry at myself and someone tries to cheer me up, I refuse it.	TPA-SD
5	If I am disappointed by myself, I do not ask for emotional support.	TPA-SD
6	If I could help an unfriendly person with a problem, I don't do it.	TPA-OD
7	If I am sad, I refuse to participate in activities that could cheer me up.	TPA-SD
8	If I am angry at someone, I will not provide that person with emotional support.	TPA-OD
9	When I am going out with a group, I do not say what I would like to do.	TPA-SD
10	If I planned to make someone a gift and I am disappointed by that person, I will not give him/her the gift.	TPA-OD
11	If I can withhold something good from someone who has hurt me, I will do it.	TPA-OD
12	If someone has hurt me, I refuse to help that person with making difficult decisions.	TPA-OD
13	When I realize that I am not good at a game, I get frustrated and stop trying to win.	TPA-SD
14	When I am angry with my partner, I exclude him/her from nice activities.	TPA-OD
15	If I cannot manage to do something, I do not seek help, because in this case I deserve to fail.	TPA-SD
16	When I'm mad at someone, I do not do anything bad to him/her, but I also stop doing anything good.	TPA-OD

17	If I am supposed to give nice greetings to someone I do not like, I refrain from doing so.	TPA-OD
18	If I feel sad, I throw myself into work instead of doing something pleasant.	TPA-SD
19	When someone commends on me, I am convinced that person just wants to be nice.	TPA-SD
20	If a person superior to me at work annoys me, I reduce my involvement in our teamwork.	TPA-OD
21	If I have done something, I am ashamed of, I don't deserve anything nice afterwards.	TPA-SD
22	If I am annoyed by someone, I refuse to give that person credit even if they deserve it.	TPA-OD
23	Even when I am exhausted from work, I do not give myself a break.	TPA-SD
24	When I am angry with someone, I try to spoil nice thing for that person.	TPA-OD
25	If my partner does not see my needs, I pay him/her back by, for example, doing the shopping or cooking just for me.	TPA-OD
26	If I am successful at a task, I spoil that success for me afterwards.	TPA-SD
27	When I'm feeling bad, I do not allow myself things that would be good for me.	TPA-SD
28	When someone hurts me, I revenge myself by not calling that person's attention to the fact that they made a mistake, forgot an appointment, or had some other mishap.	TPA-OD
29	When I am very stressed at work, I still do not allow myself a break.	TPA-SD
30	If a friend has hurt me, I purposely let him/her feel it by reducing the contact.	TPA-OD
31	If I could help someone I do not like, I will not do it.	TPA-OD
32	Even when I am sad, I still care more about others than about myself.	TPA-SD

Note. OD = other-directed; SD = self-directed.

Item reduction

Four items of the 16-items TPA-OD scale were removed due to items difficulties below .20 (items 14, 24, 25, and 28). No item had to be removed due to inter-item correlations. All remaining items demonstrated factor loadings above .30 on the one-factor solution of a principal axis factoring. Six items of the 16-items TPA-SD scale were removed due to an item difficulty below .20 (items 1, 15, 21, 26, 27, and 29). Again, no item had to be removed due to inter-item correlations. Two items were removed because of factor loading below .30 on the one-factor solution of a primary axis factoring (items 13 and 32).

Model-fit of the two-factor solution

The model fit of the bi-factorial solution of the refined 32-items version of the TPA was acceptable. The *Chi-square-df-ratio* was 1.69, *RMSR* = .08 and *fit.off* = .92. Factor loadings of

all items on the respective factor (other-directed or self-directed passive aggression) ranged between .33 and .75. TPA-OD and TPA-SD demonstrated a medium-sized intercorrelation, r = .39.

Revision of the TPA

Based on these results the authors (CGS, ME, SKS, HKM, and TM) revised the items of the TPA and added another 4 items for the validation study (i.e., 18 items per scale).

Supplement B. Item characteristics of the 36-item version of the Test of Passive Aggression

Table S2. *Item characteristics of the 36-item version of the Test of Passive Aggression*

Item	Scale	*n*	*M*	*SD*	skew.	kurt.	diff.	Pre-reduction item-total correlation	Post-reduction item-total correlation
1	TPA-SD	307	3.33	1.31	-0.38	-1.03	.58	.34	
2	TPA-SD	307	2.24	1.19	0.55	-0.81	.30	.48	.42
3	TPA-OD	307	2.75	1.25	0.19	-1.04	.42	.45	.45
4	TPA-SD	307	2.06	1.22	0.87	-0.50	.26	.39	
5	TPA-OD	307	2.47	1.18	0.40	-0.71	.36	.45	.46
6	TPA-OD	307	2.30	1.16	0.65	-0.40	.33	.24	
7	TPA-OD	307	2.74	1.37	0.28	-1.13	.44	.09	
8	TPA-SD	304	2.77	1.26	0.21	-0.88	.44	.01	
9	TPA-SD	305	2.52	1.24	0.39	-0.83	.38	.38	.40
10	TPA-SD	307	2.38	1.23	0.46	-0.92	.35	.54	.53
11	TPA-OD	306	2.40	1.22	0.51	-0.66	.35	.42	.39
12	TPA-SD	307	3.05	1.24	-0.09	-0.93	.51	.42	.43
13	TPA-OD	307	3.00	1.20	0.03	-0.92	.49	.56	.58
14	TPA-SD	306	3.17	1.41	-0.22	-1.14	.55	.41	.42
15	TPA-SD	305	2.84	1.32	0.19	-1.05	.46	.54	.56
16	TPA-SD	305	3.36	1.26	-0.32	-0.84	.57	.53	.53
17	TPA-SD	307	2.57	1.20	0.44	-0.73	.39	.59	.61
18	TPA-SD	305	2.83	1.31	0.11	-1.05	.47	.56	.55
19	TPA-OD	306	3.07	1.20	-0.07	-0.92	.50	.50	.49
20	TPA-OD	307	3.12	1.22	-0.17	-0.92	.52	.46	.49
21	TPA-SD	306	2.87	1.23	0.07	-0.86	.47	.26	
22	TPA-OD	307	2.92	1.07	0.07	-0.48	.47	.59	.61
23	TPA-OD	305	3.06	1.21	-0.02	-0.88	.49	.48	.52
24	TPA-OD	307	2.10	1.13	0.90	0.06	.28	.41	
25	TPA-OD	307	2.73	1.09	0.19	-0.49	.43	.14	
26	TPA-OD	307	2.86	1.25	0.06	-0.98	.47	.26	
27	TPA-OD	307	2.54	0.94	1.97	1.97	.14	.35	
28	TPA-SD	307	3.65	1.31	-0.75	-0.54	.66	.35	
29	TPA-OD	307	2.14	1.09	0.75	-0.20	.30	.43	.44
30	TPA-SD	306	3.14	1.34	-0.24	-1.12	.53	.59	.59
31	TPA-SD	307	3.26	1.24	-0.09	-1.02	.57	.32	
32	TPA-SD	307	2.88	1.37	0.02	-1.20	.47	.49	.51

33	TPA-OD	304	2.84	1.31	0.10	-1.08	.48	.41	.44
34	TPA-OD	306	2.52	1.23	0.30	-0.61	.35	.54	.53
35	TPA-SD	307	2.59	1.31	0.38	-0.98	.40	.55	.56
36	TPA-OD	307	3.00	1.21	0.00	-0.82	.50	.48	.50

Note. Item numbers reflect the item number in the 36-item version of the Test of Passive Aggression; TPA-SD = self-directed aggression scale; TPA-OD = other-directed aggression scale; SD = standard deviation, skew. = skewness; kurt. = kurtosis; diff. = difficulty; item-total correlations were analysed separately for both scales. Pre-reduction item-total correlation = item-total correlations of each scale before item reduction. Post-reduction item-total correlation = item-total correlations of each scale after item reduction.
